# Recycling of Spent Pot Lining First Cut from Aluminum Smelters by Utilizing the Two-Step Decomposition Characteristics of Dolomite

**DOI:** 10.3390/ma13225283

**Published:** 2020-11-22

**Authors:** Yifei Wang, Xiping Chen, Shaojun Zhang, Peixu Yang

**Affiliations:** School of Material Science and Engineering, Zhengzhou University, Zhengzhou 450001, China; 13676996694@163.com (Y.W.); zhangshaojun@zzu.edu.cn (S.Z.)

**Keywords:** Spent Pot Lining First Cut, dolomite, Pidgeon Process, recycling

## Abstract

Spent Pot Lining First Cut (shortened to SPL-1cut) is a solid waste discharged from a primary aluminum electrolytic production process. SPL-1cut is classified as hazardous waste in China because it contains large amounts of soluble sodium fluoride and a tiny amount of cyanide. Most of SPL-1cut is carbon—about 65%—and its calorific value is 22.587 MJ∙kg^−1^. There is a high level of sodium fluoride in SPL-1cut—about 15%—and sodium fluoride is randomly distributed in the carbon granule. The recycling of SPL-1cut using dolomite as a reactant, based on the characteristics of the two-step decomposition of dolomite at a high temperature, is discussed. The recycling of SPL-1cut was performed under the following heating conditions: the heating temperature was 850 °C, the holding time was 120 min, and 40% of the dolomite was added to the SPL-1cut. It was found that the cyanides are completely oxidized and decomposed, and dolomite is decomposed into MgO and active CaCO_3_. At the same time, NaF reacts with active CaCO_3_ and converts into CaF_2_. The results provide references for using SPL-1cut as an alternative fuel in the dolomite calcination process of the Pidgeon Process.

## 1. Introduction

The alumina-cryolite molten salt electrolysis process is currently the most mature industrial-scale primary aluminum production method and is widely used throughout the world. The production of primary aluminum from molten salt electrolysis has brought about some environmental problems, among which spent pot lining (SPL) is one of the most prominent. The service life of aluminum electrolytic cells is generally 5–8 years [1,2]. About 30 kg of SPL is discharged per tonne of primary aluminum produced [2]. SPL is mainly composed of first cut materials (carbon-rich) and second cut materials (refractory-rich), of which SPL first cut (shortened to SPL-1cut) account for about 30–50% of the total mass [3]. Per tonne of primary aluminum, about 10–15 kg of SPL-1cut is generated as a by-product [4]. SPL-1cut is the most major hazardous waste from smelters, mainly containing carbon and sodium fluoride. SPL-1cut contains a large fraction of carbon (about 65%); a high level of sodium fluoride (about 15%); and small amounts of cryolite, calcium fluoride, alumina, and other substances [5]. SPL-1cut contains potential carbon energy and fluorine, which has important utilization value [6,7]. How to effectively recycle SPL-1cut has become a prominent problem which is being focused upon by the whole aluminum industry. In recent years, the rapid development of metallurgy, material design, and functionalization has provided a strong support for the recycling of industrial waste [8,9,10].

The toxicity of SPL-1cut is mainly due to soluble fluorides and cyanides. The content of fluorides in SPL-1cut is relatively high, and the most common type of them are sodium fluoride. Sodium fluoride is easily soluble in water, causing serious pollution to water resources and soils and easily forming high-fluorine water and high-fluorine soil, which in turn endangers the growth and survival of animals and plants. When cyanides meet water, they will release the harmful gas NH_3_. Heating at a high temperature is the simplest and most effective way to eliminate cyanides, which begin to decompose when heated to above 400 °C [11,12]. In general, by adding a certain amount of CaCO_3_ in a high-temperature environment, the soluble NaF in SPL can be converted into stable CaF_2_, reducing the harm of soluble fluoride sharply. In previous studies, limestone was used to convert harmful fluorides into harmless and stable fluorides [11,13,14]. However, when limestone is used as a reactant to turn soluble fluorides into stable fluorides at a high processing temperature, some negative effects appear, such as the final product easily agglomerating and adhering to the furnace lining, so an anti-adhesive additive needs to be added. Reynolds Metals Company disclosed a method for treating SPL at a temperature of 650–930 °C using limestone and adding a certain amount of metal silicates [13]. Therefore, it is necessary to find a new reactant to treat SPL.

Dolomite is a kind of natural ore, and its main component is CaMg(CO_3_)_2_. Dolomite, after crushing and calcining, is an important raw material for the silicon thermal process of magnesium (the Pidgeon Process). MgCO_3_ and CaCO_3_ in dolomite exists in the structure of double salt with a strong binding force [15], and its decomposition temperature is higher than that of the CaCO_3_ in limestone. It is interesting that dolomite has the characteristics of two-step decomposition at high temperatures and fluorite is used as a catalyst in the Pidgeon Process [16,17]. CaF_2_ is mainly derived from natural fluorite, which is scarce in China. The use of natural fluorite is one of the reasons for the high cost of the Pidgeon Process. Based on the characteristics of the SPL-1cut and dolomite, some interesting experiments are put forward. What will happen if a certain amount of dolomite is added to the SPL-1cut at a high temperature? The following experimental results are expected: CaCO_3_ in the dolomite can convert fluorides into CaF_2_; toxic and harmful substances such as cyanides, nitrides, and carbides in the SPL-1cut can be eliminated at high temperatures; at the same time, the carbon in the SPL-1cut is fully burned at high temperatures to generate heat and provide reaction energy.

## 2. Composition and Performance of SPL-1cut

**Figure 1:**
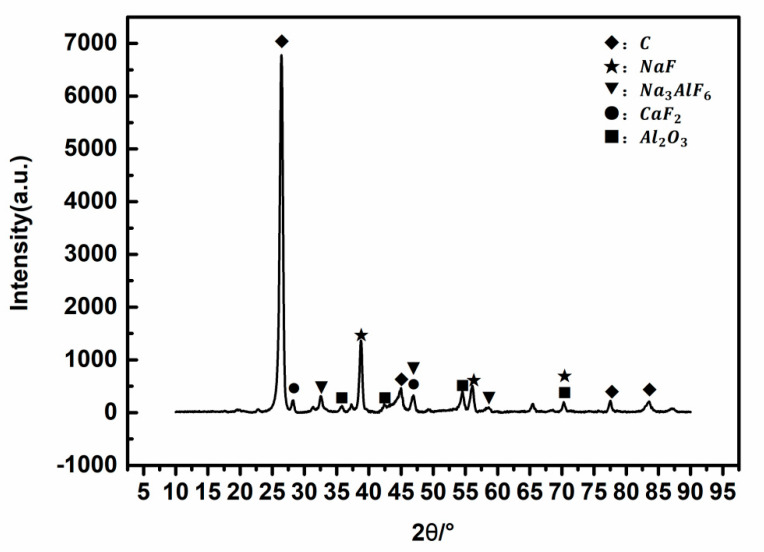
XRD pattern image of Spent Pot Lining First Cut (SPL-1cut).

**Figure 2:**
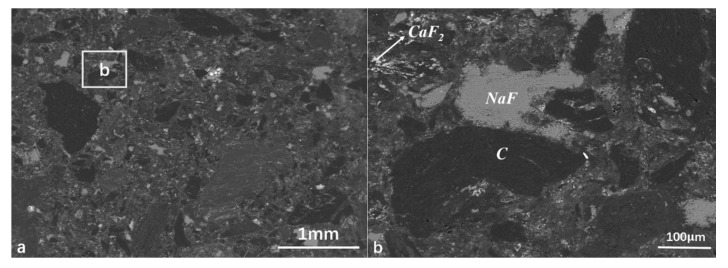
SEM-EDS analysis of SPL-1cut. (**a**) the microstructure of SPL-1cut; (**b**) enlarged view of the b area.

The SPL-1cut samples used in the experiments were taken from the newly shut-down aluminum reduction pots, which belong to three different smelters located in northwest, southwest, and central China with line currents of 400–500 kA. The service age of these pots was between 5 and 7 years. Three kinds of SPL-1cut were crushed by a hammer crusher s (SP-200, Tianyuan Instrument, Hebi, China) and fully mixed according to the mass ratio of 1:1:1, and then the mixed sample was ground by a ball mill and stored in an airtight plastic bucket for subsequent tests and experiments. X-Ray Diffractomer (XRD) (XRD-6100, SHIMADZU CORPORATION, Kyoyo, Japan), scanning electron microscope (SEM) (FEI-Quanta-250, FEI Quanta, Hillsboro, OR, USA), a calorimeter (5E-C5500,Changsha Kaiyuan Instruments, Changsha, China), and a sulfur analyzer (5E-AS3200B, Kaiyuan Instruments, Changsha, China) were used for identifying the physical and chemical properties of SPL-1cut. The phase composition of SPL-1cut is shown in Figure 1, and the micro-morphology is shown in Figure 2. 

The matrix of the SPL-1cut is carbon and contains sodium fluoride, calcium fluoride, alumina, cryolite, and other components (as shown in Figure 1). The content of sodium fluoride is the highest among fluorides. Sodium fluoride has a high abundance and a wide-scale distribution in SPL-1cut (as shown in Figure 2a). There is a clear boundary between sodium fluoride and the carbon matrix (as shown in Figure 2b), so sodium fluoride and carbon can be initially separated by suitable processing.

The calorific value and sulfur content are, respectively, listed in Table 1 and Table 2.

The calorific value of the SPL-1cut is relatively high, with an average value of 22.587 MJ∙kg^−1^, which is about 77.9% that of standard coal (as shown in Table 1). During the test, it was found that some white solid substances adhered to the inner wall of the test crucible and some gray solid substances were taken out of crucible. The reason for this phenomenon is that SPL-1cut contains a certain amount of non-combustible components such as fluorides. A small amount of fluoride-carbon mixture was taken out by gas flow when the CO_2_ gas rapidly escaped from the SPL-1cut, but the fluorides condensed again on the inner wall of the crucible during the combustion. The calorific value of SPL-1cut has good reproducibility via using aluminum foil to coat the sample. 

The content of sulfur in SPL-1cut is relatively low (as shown in Table 2). When SPL-1cut is burned as an alternative fuel, the SO_2_ emissions will be sharply reduced and cause a small harmful impact on the regional environment.

The dolomite used in the experiment is a high-quality dolomite ore which was taken from Shanxi Province, China. It was processed by a jaw crusher (EP-II, Qitian Instrument, Hebi, China) and a ball mill (BYT-XGB4, Boyuntong Instruments, Nanjing, China) to obtain a suitable powder. The chemical components of dolomite are shown in Table 3.

## 3. Experimental

SPL-1cut and dolomite were crushed and sieved separately, mixed according to a certain ratio, and loaded into corundum crucibles (70 × 70 × 50, Boyuntong Instruments, Nanjing, China), then crucibles with the experimental sample were placed in an ultra-high-temperature muffle furnace (QSH-1700M, Boyuntong Instruments, Nanjing, China) for heat treatment. The purpose of the experiments was mainly to eliminate cyanides in SPL-1cut and to obtain a high conversion ratio of fluoride. The experimental temperature was 750–950 °C, the particle size of the sample was in the range of 0–200 mesh, the residence time was 90–210 min, and the mass ratio of dolomite in the experimental sample was from 10 to 50 wt%. The influences of the temperature, the particle size of the sample, the residence time, and the weight ratio of dolomite on the reaction loss ratio were studied.

The main reactions during the heat treatment were the combustion of carbon and the reactions between fluoride and dolomite (as shown in reactions 1, 2, 3, 4).

C + O_2_ = CO_2_ (1)

CaMg(CO_3_)_2_ = CaCO_3_ + MgO + CO_2_ (2)

CaMg(CO_3_)_2_ + 2NaF = CaF_2_ + Na_2_CO_3_ + MgO + CO_2_ (3)

6CaMg(CO_3_)_2_ + 2Na_3_AlF_6_ = 6CaF_2_ + 3Na_2_CO_3_ + 6MgO + Al_2_O_3_ + 9CO_2_ (4)

The combustion of carbon not only causes a large loss of the total weight of the sample but also causes more closer contact between fluoride and CaCO_3_, which helps promote the conversion of fluoride.

In order to identify which condition is better, the reaction loss ratio Y is used to visually judge the experimental results.

Y = (g_1_ − g_2_)/g_1_ × 100% (5)

where g_1_ is the initial weight of the mixed sample and g_2_ is the weight of the final product after processing.

## 4. Results

**Figure 3:**
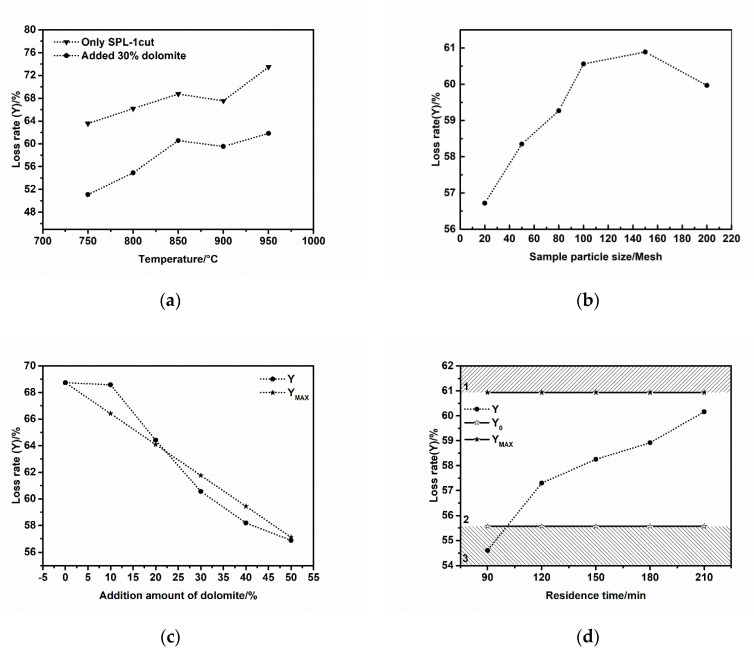
Trend of reaction loss ratio under different experimental conditions ((**a**) 100 mesh, 30 wt% dolomite, 180 min; (**b**) 850 °C, 30 wt% dolomite, 180 min; (**c**) 850 °C, 100 mesh, 180 min; (**d**) 850 °C, 40 wt% dolomite, 100 mesh).

Different experiments were carried out under different experimental conditions according to the preset experimental scheme. Experimental results are shown in Figure 3. In order to facilitate the analysis of the experimental results, we set Y_0_ as the optimal loss ratio, which only includes the carbon burning loss and the decomposition loss of magnesium carbonate in dolomite during the thermal treatment; Y_MAX_ is the maximum loss ratio, which is the complete combustion loss ratio of SPL-1cut plus the complete decomposition loss ratio of dolomite. SPL-1cut did not react with the dolomite during the process.

Y_0_ = [(g_1_ × c_1_ × Y_1_)+ (g_1_ × c_2_ × Y_3_)]/g_1_ × 100% (6)

Y_MAX_ = [(g_1_ × c_1_ × Y_1_)+ (g_1_ × c_2_ × Y_2_)]/g_1_ × 100% (7)

where c_1_ is the weight fraction of SPL-1cut in the mixed sample, c_2_ is the weight fraction of dolomite in the mixed sample, Y_1_ is the loss ratio when SPL-1cut is separately processed, Y_2_ is the loss ratio when dolomite is separately processed, and Y_3_ is the loss ratio when the first decomposition step of dolomite occurs—that is, only the MgCO_3_ in dolomite is completely decomposed into MgO.

It can be seen clearly from Figure 3a that the loss ratio of SPL-1cut reaches a steady state at 850 °C. When the temperature rises to 900 °C, the electrolyte components in the SPL-1cut become eutectic and cause the mixture to become sticky, resulting in the insufficient combustion of the carbon in SPL-1cut, and the loss ratio decreases slightly. When the temperature is increased to 950 °C, the low-melting-point electrolyte component in SPL-1cut may decompose and generate volatile loss, resulting in a large increase in the loss ratio. The loss ratio change trend of the experimental sample mixed with 30 wt% dolomite is similar to that of SPL-1cut burned alone, and is basically stable at 850 °C. The melting point of Na_2_CO_3_ in the reaction product is 851 °C. At 900 °C, Na_2_CO_3_ easily melts and the mixture becomes sticky, causing a small decrease in the loss ratio. The addition of dolomite achieves the purpose of converting fluoride into CaF_2_, and prevents a large increase in the loss ratio when the temperature is up to 950 °C. However, due to the inadequate amount of dolomite, the fluoride was not completely converted.

The loss ratio of the experimental sample becomes larger and larger when the particle size of the sample becomes finer and finer. The maximum value can be obtained when the particle size of the sample is finer than 150 mesh. As the particle size continues to become finer than 200 mesh, the loss ratio significantly decreases (as shown in Figure 3b). The reason for this phenomenon is that over-fine samples are much more likely to cause adhesion and agglomeration at a high temperature, which causes negative effects on all reactions and then reduces the loss ratio. The particle size of SPL-1cut has a certain influence on the subsequent treatment reaction. Similar to the size effect and the quantum confinement effect of materials, it has been widely studied and applied in the field of nano materials [18,19,20].

As shown in Figure 3c, the actual loss ratio Y in the upper left part of the graph is greater than Y_MAX_. In this region, SPL-1cut and dolomite did not react or incompletely reacted, and the amount of dolomite added was insufficient. In the lower right part of the graph, the actual loss ratio Y is less than the maximum loss ratio Y_MAX_. In this area, the SPL-1cut reacted with the dolomite and the reaction finished completely.

The fixed experimental conditions in Figure 3d were that the temperature was 850 °C, the particle size was less than 100 mesh, and the dolomite weight ratio was 40 wt%. According to the change trend of reactions in Figure 3d, it can be divided into three areas: area 1, no reactions between the SPL-1cut and dolomite; area 2, the carbon in SPL-1cut burned completely, the MgCO_3_ in the dolomite also decomposed completely, and the reaction between fluoride and CaCO_3_ was thoroughly completed; area 3, the reaction was incomplete. During the processing of SPL-1cut, all reactions will be more thorough when the actual loss ratio Y is between Y_0_ and Y_MAX_.

## 5. Discussion

**Figure 4:**
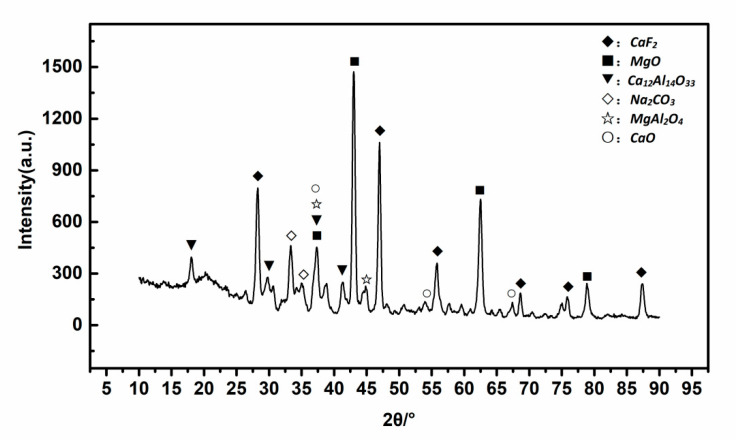
XRD pattern of the final product.

**Figure 5:**
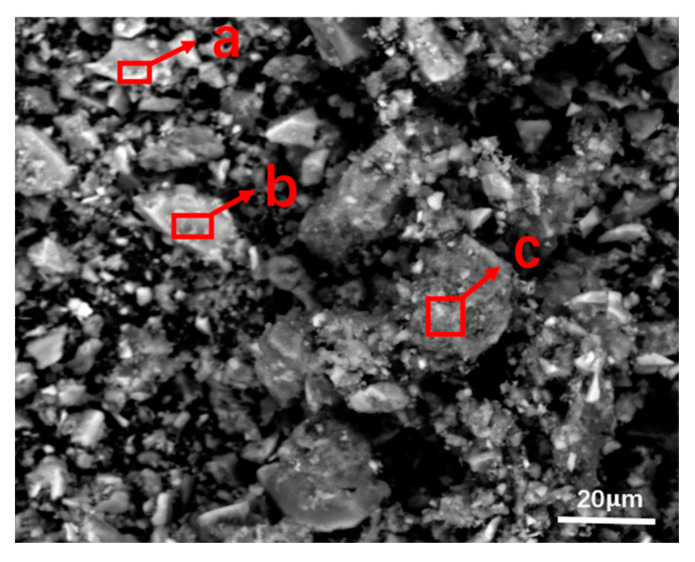
SEM-EDS analysis of the final product. Micro area (**a**) shows conversion reaction of fluoride; micro area (**b**) shows complete conversion of fluoride; micro area (**c**) shows dolomite decomposition.

XRD and SEM-EDS were used to analyze the final product, which was produced at 850 °C by keeping the sample size finer than 100 mesh, the dolomite addition ratio at 40 wt%, and the residence time at 120min. The results are shown in Figure 4 and Figure 5.

**(1) EDS analysis of different positions of the final product (wt%).**

**Table:** 

Elements	C	F	Na	O	Ca	Mg	Al	K	Si
**a**	6.75	6.81	9.52	32.42	28.05	1.09	6.48		8.9
**b**	4.87	18.46	17.16	21.5	26.61	0.65	0.7	0.31	9.72
**c**	9.42	15.77	11.48	29.94	2.69	26.59	1.34	0.78	

**(2) EDS analysis of different positions of the final product (At%).**

**Table:** 

Elements	C	F	Na	O	Ca	Mg	Al	K	Si
**a**	12.05	7.69	8.88	43.47	15.01	0.96	5.15		6.8
**b**	8.93	21.41	16.45	29.61	14.63	0.59	0.57	0.18	7.63
**c**	14.8	15.67	9.43	35.32	1.27	22.2	0.93	0.38	

MgCO_3_·CaCO_3_ in dolomite exists in a double salt form whose high-temperature decomposition process can be divided into two steps. The decomposition of MgCO_3_ occurs first, and then CaCO_3_ decomposition occurs. This characteristic of dolomite ensures that it can provide effective, active CaCO_3_ as a reactant when reacting with fluorides in SPL-1cut. It can be clearly seen from Figure 4 that the final product mainly consists of CaF_2_ and MgO. In Figure 5, area a is a conversion reaction place of fluoride, and its main substances are CaCO_3_ and NaF; area b is a complete conversion place of fluoride, and its main substances are CaF_2_ and Na_2_CO_3_; the main substances in area c are MgO and NaF, and the first step of dolomite decomposition occurred in this area. During thermal processing, MgCO_3_·CaCO_3_ in dolomite was decomposed to generate MgO and CaCO_3_, and the newly generated CaCO_3_ reacted with NaF to ensure the conversion of fluorides. Furthermore, along with the temperature rises, carbon combustion, and dolomite first-step decomposition, reactions between fluorides and calcium carbonate occur in turn. When dolomite is used to treat SPL-1cut, the final product containing MgO and CaF_2_ can be more easily recycled in the Pidgeon Process. The process has more practical value compared with the previous process, which used limestone. 

When the SPL-1cut was processed for 120 min under the conditions of 850 °C, particle size finer than 100 mesh, and a dolomite addition fraction at 40 wt%, the complete conversion of fluorides was achieved by fluorides reacting with newly generated CaCO_3_. At the same time, the MgCO_3_ in dolomite was decomposed into MgO. In a word, the processing of SPL-1cut is feasible by adding dolomite. The process has the following advantages: the temperature is not high, the concentration of soluble fluoride in the final product can be reduced to a safe limit, and the final product does not show an obvious adhesion phenomenon.

The leaching toxicity of the SPL-1cut final product (treated with dolomite) was measured, and the results are listed in Table 4. 

It can be seen from Table 4 that F^−^ and CN^−^ in the final product are lower than the national limit (GB5085.3-2007) [21]. 

## 6. Conclusions

The recycling of SPL-1cut is achieved by adding dolomite to SPL-1cut based on the characteristics of the two-step decomposition of dolomite at high temperatures. During the thermal process, the CaCO_3_ newly generated by the first-step decomposition of dolomite will react with fluorides to generate calcium fluoride, an insoluble fluoride. The cyanides will be completely oxidized and decomposed. The exothermic combustion of carbon provides enough heat for other reactions.

Dolomite is used as a reactant to reduce the soluble fluorides in SPL-1cut. The final product is mainly composed of MgO and CaF_2_. It can be used as a raw material and as an alternative fuel for magnesium production by the Pidgeon Process so as to save fluorite and standard coal.

The recycling feasibility of SPL-1cut in magnesium production by the silicon thermal process is discussed in this paper, and some preliminary results were obtained. In the future, continuous research will be conducted on the following aspects: the addition of SPL-1cut instead of standard coal for the dolomite calcination process, including the blending ratio of SPL-1cut, and the influences of SPL-1cut on the magnesium reduction tank and magnesium yield.

## Figures and Tables

**Table 1:** The calorific value of SPL-1cut (MJ∙kg^−1^).

Parallel Samples	Not Coated	Coated
**1**	21.389	22.718
**2**	22.099	22.359
**3**	21.670	22.683

**Table 2:** Sulfur content of SPL-1cut (%).

Parallel Samples	SPL-1cut
**1**	0.16
**2**	0.16
**3**	0.15

**Table 3:** Chemical composition of dolomite.

Chemical Composition	CaO	MgO	Al_2_O_3_	Fe_2_O_3_	CO_2_
**Content (%)**	31.22	22.31	0.05	0.05	47.04

**Table 4:** Leaching toxicity of the SPL-1cut final product treated with dolomite (mg∙L^−1^).

Parallel Samples	F^−^	CN^−^
**1**	47.53	0.047
**2**	49.83	0.051
**3**	48.37	0.048
